# Genetic determination of the enhanced drought resistance of rice maintainer HuHan2B by pedigree breeding

**DOI:** 10.1038/srep37302

**Published:** 2016-11-17

**Authors:** Haibin Wei, Fangjun Feng, Qiaojun Lou, Hui Xia, Xiaosong Ma, Yunhua Liu, Kai Xu, Xinqiao Yu, Hanwei Mei, Lijun Luo

**Affiliations:** 1 Shanghai Agrobiological Gene Center, Shanghai 201106, China

## Abstract

The ongoing deficit of fresh water resource in rice growing regions has made the selection of water-saving and drought-resistance rice (WDR) a crucial factor in developing sustainable cultivation. HuHan2B, a new *japonica* maintainer for WDR breeding, had the same yield potential as recurrent parent HanFengB but showed improved drought resistance in fields. We investigated the genomic content accumulation and candidate genes passed from parent to offspring using the genomic and transcriptomic approaches. The genomic constitution indicated that the genetic similarity was 84% between HuHan2B and HanFengB; additionally, 7,256 genes with specific alleles were inherited by HuHan2B from parents other than HanFengB. The differentially expressed genes (DEGs) under drought stress showed that biological function was significantly enriched for transcript regulation in HuHan2B, while the oxidation-reduction process was primarily enriched in HanFengB. Furthermore, 36 DEGs with specific inherited alleles in HuHan2B were almost involved in the regulatory network of TFs and target genes. These findings suggested that major-effect genes were congregated and transformed into offspring in manner of interacting network by breeding. Thus, exploiting the potential biological function of allelic-influencing DEGs would be of great importance for improving selection efficiency and the overall genetic gain of multiple complex traits.

Rice is one of the most important crops for global food security and sustainable agriculture development^1^. The domestication of cultivated rice (*Oryza sativa L*.) throughout the history of human civilization has produced many superior plant phenotypes that have been improved via conventional plant breeding practices to accommodate the needs of farmers and consumers. Improving grain yield remains one of the most important goals of crop breeding; however, current paddy rice cultivation depends on an ample water supply and is more vulnerable to drought stress than other cropping systems^2^. Water deficit is an important environmental constraint affecting rice physiological processes that are involved in its growth and development, as well as agricultural productivity^3^. Despite global efforts to obtain large amounts of fresh water resources, water-saving and drought-resistance rice (WDR) breeding is economically important for crop production and food security^4^,^5^.

Due to biotechnology developments, recent breakthroughs in understanding the molecular mechanisms of abiotic stress responses have accelerated the determination of the complex traits underlying variations in stress tolerance in crop plants. The effects of drought stress on rice are being studied at the molecular, physiological and biochemical level^6^,^7^. Many genes have been found to activate a series of complicated regulatory mechanisms to enhance drought resistance, such as *DRO1*^8^, *OsNAC5*^9^,^10^, and *OsGL1-6*^10^. Moreover, many of these genes with diverse functions are induced or repressed by drought stress. Gene networks have also been used to elucidate the functions of genes and to determine how various genes govern drought-stress response regulation^11^,^12^. Additionally, the availability of deep sequencing technologies accelerated the process of identifying stress-inducible genes at the whole-genome level in rice^13^,^14^,^15^. Although an increasing number of genes related to drought resistance has been isolated and identified, determining the mechanisms remains a challenging task because it involves multiple metabolic and morphologically adaptive pathways^16^.

Breeding has been regarded as a reliable method for selecting excellent valuable agronomic traits for rice, and genetic materials from rice parents were convenient in studying the gene actions involved in drought stress response. We bred a new *japonica* maintainer HuHan2B for WDR breeding, and its cytoplasmic male sterile (CMS) line Huhan 2A was hybridized with restorer line Xiangqing to breed a hybrid combination Hanyou 8, which was registered in China in 2010 and the extensive areas were increased to 12,000 hectares in 2015^17^,^18^. HuHan2B was developed using a backcross breeding procedure that introduced drought resistance into recurrent parent HanFengB. The agronomic traits of HuHan2B were identified in previous studies, and the source of genetic variation was involved in a clear breeding pedigree^17^,^19^. The molecular genetic basis of drought resistance during WDR breeding has been unknown; thus, it is necessary to investigate the genetic mechanisms conferring adaptation to water saving and drought resistance in HuHan2B.

In the present study, five varieties of HuHan2B pedigree breeding were used as materials to study the genetic constitution in HuHan2B via genome resequencing. The differentially expressed genes (DEGs) in the leaves were analyzed using transcriptomic sequencing of HuHan2B and recurrent parent HanFengB under drought stress. We further detected candidate genes in combined with genomic and transcriptomic data and discovered they were congregated into the gene network to be transformed into offspring by breeding. Based on these findings, we presented a transcriptional regulation model of response to drought stress as determined by TFBS complexity. This research is essential and helpful for understanding the manner in which genetic variations of drought resistance traits are passed from parent to offspring following artificial selection by pedigree breeding.

## Results

### Whole genome resequencing

The genomic DNA from HuHan2B pedigree breeding varieties was sequenced via Illumina HiSeq 2000. A total of 0.5 billion paired reads of raw data were obtained, generating approximately 0.47 billion high-quality clean reads after removing adaptor sequences, duplications and low-quality reads (Table 1). Mapping the clean reads to the Nipponbare reference genome (MSU 7.0) revealed that the mapping rate ranged from 95.51% to 98.38%, while the sequencing depth across the five rice varieties ranged from 5.3-fold to 51.7-fold. An increased sequencing depth produced greater coverage of genes (Table 1). We detected at least a 1-fold coverage in the gene coding region (ranging from 76.3% to 99.26% among five varieties, Table 1), which enabled sufficient coverage to perform variant calling.

SNPs and genotypes were called using the Unified Genotyper of the Genome Analysis Toolkit (GATK), which enabled the joint analysis of all samples from one population. In total, we obtained 1.1 million high-quality SNPs. In this study, we evaluated the accuracy of SNP calling using four independent varieties via Illumina RiceSNP60 whole-genome SNP array. After excluding missing data in either the SNP calling or SNP array, approximately 16,000 effective homozygosis sites were shared among the two datasets. Accuracy validation indicated that more than 99% of the SNPs were in accordance with the physical positions and genotypes. The SNP discordance rates between the two datasets are presented in Table S1.

### SNPs distribution in the Nipponbare reference genome

The distributions of SNPs across the Nipponbare reference genome in different gene coding regions were assessed (Table S2). The number of SNPs was the greatest on chromosome 4 (112,536) and the least on chromosome 3 (45,581), accounting for 10.37% and 4.20% of the SNPs, respectively. SNP density varied between chromosomes. Chromosome 10 had the highest density with 4.72 SNPs per Kb. Chromosome 3 had the lowest density with 1.25 SNPs per Kb. Table S2 showed that the highest polymorphism portion was found on the intergenic regions (60.33%), followed in an order of promoter, intron, exon, UTR and splicing site regions. Notably, more SNPs were located in 3′-UTR (1.27%) than in 5′-UTR (0.82%). Moreover, more SNPs were detected on the introns (9.69%) than exons (7.87%). For exonic regions, there were 109,121 synonymous SNPs and 69,097 non-synonymous SNPs, as well as 3,843 stop-gain and 883 stop-loss mutations (Table S2). Synonymous SNPs were more abundant, and the average non-synonymous to synonymous substitution ratio (Nonsyn/Syn ratio) was 0.63 in the exonic regions. Of the 187,647 genetic differences in HuHan2B and HanFengB, approximately 9,819 non-synonymous SNPs were detected in 3,609 genes, which could have drastic effects on protein structure and therefore function.

### Genetic similarity among Huhan2B and parents

The genetic characterization was performed using 1,085,144 SNPs, and pair-wise genetic similarity indexes were calculated between the five varieties; values ranged from 0.14 to 0.86 (Table 2). The genetic similarity between HuHan2B and recurrent parent HanFengB was 0.84; HanFengB was one of the genetically closest parents to HuHan2B. The lowest similarity coefficient was observed between HuHan2B and IRAT109. Thus, after successive breeding, approximately 14% of the genomic constitution of HuHan2B could be traced to grandparental upland variety IRAT109. Since the genome of grandparental P77 is not resequenced and the genomic constitution only accounts for ~3% of the observed variation in HuHan2B, we discarded it in the subsequent data analysis.

### Reconstructed recombination events in HuHan2B pedigree breeding varieties

**Figure 1:**
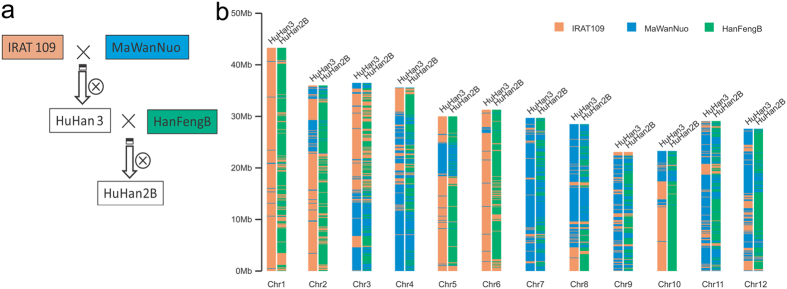
Genetic background of 5 sequenced varieties. (**a**) Pedigree of HuHan2B breeding. The male parent of each cross is listed first. The pedigree relationships assist in the planning of WDR breeding programs to enhance desirable traits. (**b**) Genome-wide distribution of genetic constitution of Huhan3 and HuHan2B. The sources of genetic regions were reconstructed by recombination events as they were derived from their parental varieties. The colors on the panels indicate the core ancestors.

To further perform the genetic characterization of genomic constitution, we evaluated genomic recombination events using a diversity-decreased level to reconstruct the HuHan2B pedigree breeding history. The genomic regions were split to represent the haplotype blocks that were descended from different parents or grandparents. Following the pedigree in Fig. 1a, the parent-offspring pairs were clearly identified by all haplotype block measures. Genomic fragments of HuHan3 included 3,315 (44.85%) haplotype blocks originating from one parent (MaWanNuo) and 4,120 (55.15%) blocks from the other parent (IRAT109) (Fig. 1b). In contrast, HuHan2B inherited 62.39% of its genomic content from the inbred line HanFengB; however, the contributions of grandparents, MaWanNuo and IRAT109, were unequal (24.83% from MaWanNuo and 12.78% from IRAT109, Fig. 1b).

### Genes with specific alleles inherited from parents other than HanFengB

Based on genotypes of pedigree breeding varieties and homologous chromosome sections from parents, we investigated the monogenic inherited characteristics of HuHan2B. We defined a specific allele in HuHan2B as the same as one of the biparental alleles but distinct from HanFengB. The origin-obtained inherited alleles (50,203) in HuHan2B were primarily consistent with homologous chromosome sections. Of these SNPs, 32,683 are located in 7,256 genes within the genebody or in proximity of a promoter. These genes with specific inherited alleles could be related to improved drought resistance performance.

### Selection of the transcriptomic analysis period under drought stress

**Figure 2:**
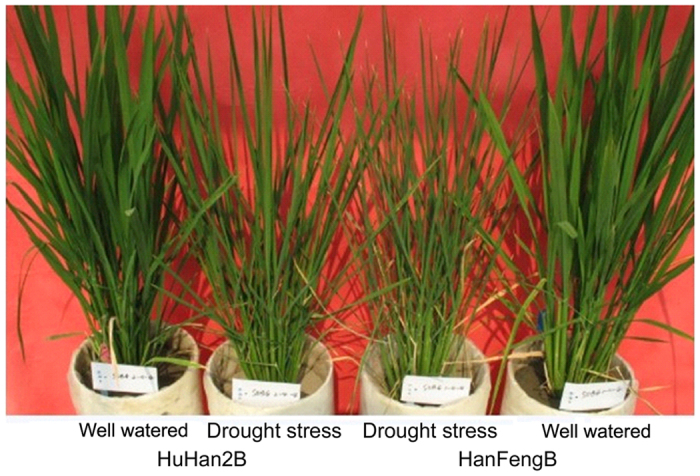
Seedling reaction of offspring HuHan2B and parental HanFengB to drought stress after 18 days of drought treatment.

**Figure 3:**
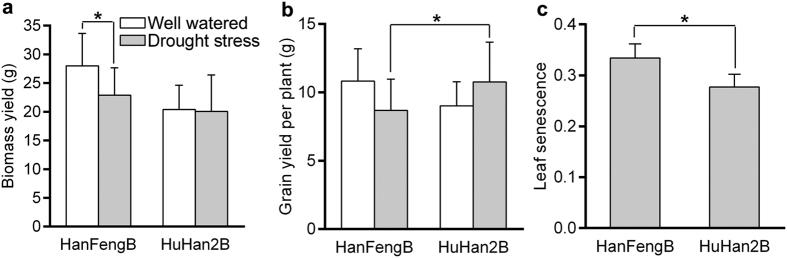
Effect of drought stress on the biomass yield, grain yield per plant, and leaf senescence of the experiment. Each value in (**a**–**c**) represents the mean ± SD of nine independent biological replicates. The * signs indicate the level of significance for the difference, *P < 0.05.

To investigate the effects of allelic diversity on drought resistance, transcriptomes of leaves were used to analyze DEGs between HuHan2B and recurrent parent HanFengB. Phenotype selection was carried out during breeding, and HuHan2B maintained the same yield potential as HanFengB but improved drought resistance (Fig. 2). The drought treatment lasted 37 days, and the soil water content was gradually decreased from 23.1% to 10.0%. When the soil water content under drought stress conditions was equivalent to half of the well-irrigated state, three sampling time points were designated (T1, T2 and T3, which corresponded to days 20, 25, and 37 after water withholding, respectively). After successive drought treatment at the later tillering stage, phenotypic evaluations of both rice varieties were conducted in maturity stages (Fig. 3). The yield components exhibited significant differences in biomass and grain weight per plant (Fig. 3a and b). The biomass yield and grain yield per plant were substantially decreased in HanFengB; however, the changes in the yields of both types were comparatively less in HuHan2B. Importantly, under drought stress conditions, the grain yield of HuHan2B was more significantly increased than that of HanFengB, and amolst equivalent to the grain yield of HanFengB under well watered conditions (Fig. 3a and b). As shown in Fig. 2, the leaves of HuHan2B looked greener than those in HanFengB. Notably, leaf senescence in HuHan2B was significantly reduced compared with HanFengB (Fig. 3c).

### Differential transcriptomic analysis between HuHan2B and parent HanFengB

**Figure 4:**
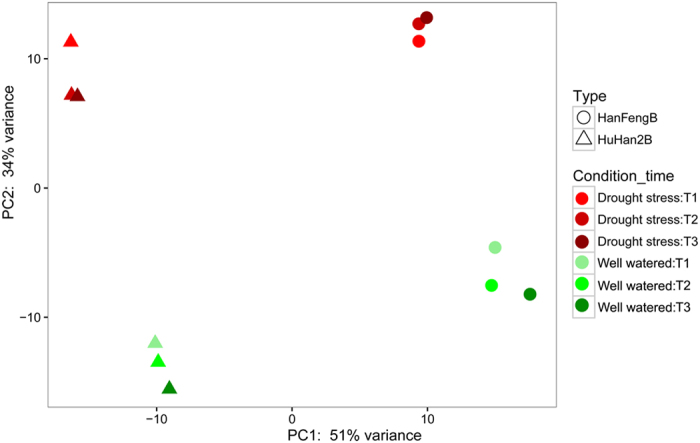
PCA analysis on 12 transcriptomes revealed genetic variation perturbations correlated with drought stress. The major changes in gene expression between the genetic variation and drought treatment were mapped onto a lower (mostly 2-dimensional) space. PC1 plotted against PC2 showed obvious differentiation between offspring HuHan2B and parental HanFengB for gene expression in response to drought stress.

A principal component analysis (PCA) was generated to detect major trends in transcriptomic data and visualized similarities among samples (Fig. 4). We observed that a clear separation was shown at the first principal component (PC1), which corresponds to varieties and accounts for ~51% of the observed variation. In contrast, the PC2, accounting for ~34%, separated samples from well watered and drought stress. Samples collected in different time points located close to each other, implying relatively less variation in gene expression by different severities of drought stress in this study.

**Figure 5:**
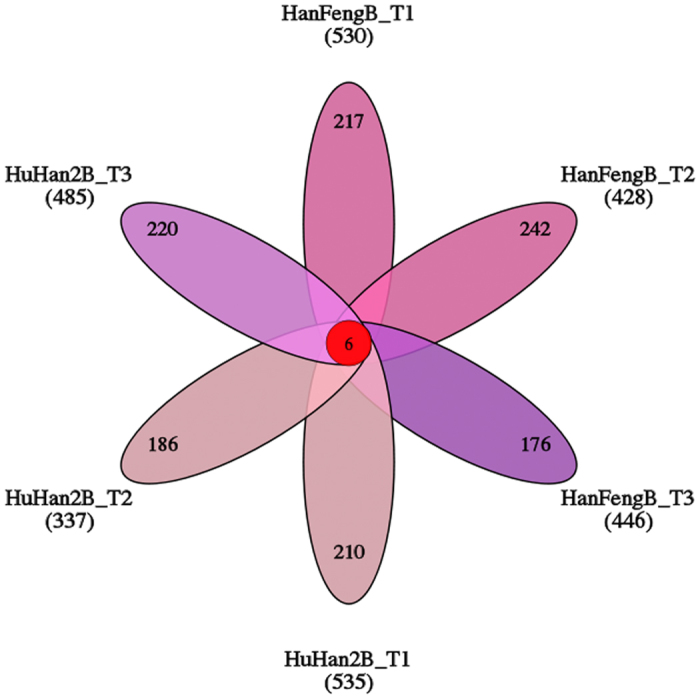
Common and specific differentially expressed genes in HuHan2B and HanFengB in three time points under well watered and drought stress conditions.

A total of 1,161 DEGs in HuHan2B and 1,197 DEGs in HanFengB were identified at three time points under well watered and drought stress conditions. Among these, 690 and 640 DEGs were up-regulated, while 613 and 590 DEGs were down-regulated, in HuHan2B and HanFengB, respectively (Table S3). The numbers of common DEGs of up-regulated and down-regulated genes were 288 and 160, respectively, in HuHan2B and HanFengB. Venn diagrams showed that a large number of DEGs were monopolized according to the various comparisons under drought stress at each time point in HuHan2B and HanFengB (Fig. 5). The larger differences in DEGs are likely due to the divergent drought-responsive regulatory mechanism in HuHan2B and HanFengB.

To obtain further insight into the significantly enriched biological processes of DEGs, gene ontology (GO) enrichment analysis was performed for each variety (Tables S4 and S5). These significantly overrepresented GO terms were primarily grouped into five functional categories: 1) Transport GO:0006810, 2) response to stimulus GO:0050896, 3) carbohydrate metabolic process GO:0005975, 4) small molecule binding GO:0036094 (molecular function), and 5) cell part GO:0044464 (cellular component). The numbers of significantly enriched GO terms were 164 and 122 in HuHan2B and HanFengB, respectively. Strikingly, 86 GO terms were shared between HuHan2B and HanFengB, involving a nearly equal amount of DEGs. Interestingly, nearly half of the GO terms were unique to HuHan2B. Furthermore, the substantial differences in the number and uniqueness of the enrichment of GO terms in each variety may reflect the differences in response to drought stress between HuHan2B and recurrent parent HanFeng.

The overview of functional categories represented the wider aspect of drought resistance; however, we sought to determine the details of the affected processes between HuHan2B and HanFengB at each time point. By specifying the deregulation cluster of DEGs at each time point, we could roughly capture the prominent processes at the various levels of drought. 1) At T1, the overrepresented GO terms with up-regulated DEGs were dominated by activities related to response to stress, abiotic stimulus, water deprivation, temperature and chemicals in both HuHan2B and HanFengB. The significant GO terms with down-regulated DEGs performed differently in both rice varieties. A few biological processes, including kinase activity, protein phosphorylation, and binding, were enriched in HanFengB but not HuHan2B. 2) At T2, the up-regulated GO terms in HanFengB included a large number of processes in cellular components, such as thylakoid part (GO: 0044436), plastid part (GO: 0044435), and chloroplast part (GO: 0044434), which are involved in the subsequent cell injury events. The down-regulated GO terms included the beta-glucan biosynthetic process. In contrast, in HuHan2B, the up-regulated GO terms continually overrepresented the response to abiotic stimulus, temperature and heat, and down-regulated GO terms had no overrepresentation. 3) At T3, the up-regulated GO terms in HanFengB continually enhanced more processes associated with the assembly and biogenesis of cellular components, such as organelle (GO:0044422) and photosynthetic membrane (GO:0034357), and the down-regulated GO terms were related to transporter activity (GO:0005215). In contrast, in HuHan2B, the up-regulated GO terms presented a different behavior: a large number of processes associated with cellular component were observed only initially, and the down-regulated GO terms were related to carbohydrate biosynthesis. Thus, the GO enrichment analysis of DEGs suggested that response to stress and carbohydrate biosynthesis were more important in response to drought stress. Compared with HanFengB, HuHan2B presented a continued increase in the expression of stress-response genes to avoid decreasing carbohydrate biosynthesis for a longer time period.

### Comparison of expression alterations and the relationship with specific inherited alleles

To further evaluate specific drought resistance response genes in HuHan2B, a filter determined the subset of DEGs by comparing the expression of HuHan2B and HanFengB under drought stress conditions at each time point. A total of 166 DEGs were identified to specifically reflect HuHan2B, while 178 DEGs were observed in HanFengB. The potential biological relevance of the genes was identified using GO enrichment analysis. These significantly overrepresented GO terms were primarily grouped as the regulation of transcription in HuHan2B (GO:0006355, ratio: 20/166). In contrast, the GO term oxidation-reduction process was primarily enriched in HanFengB (GO:0055114, ratio: 21/178).

**Figure 6:**
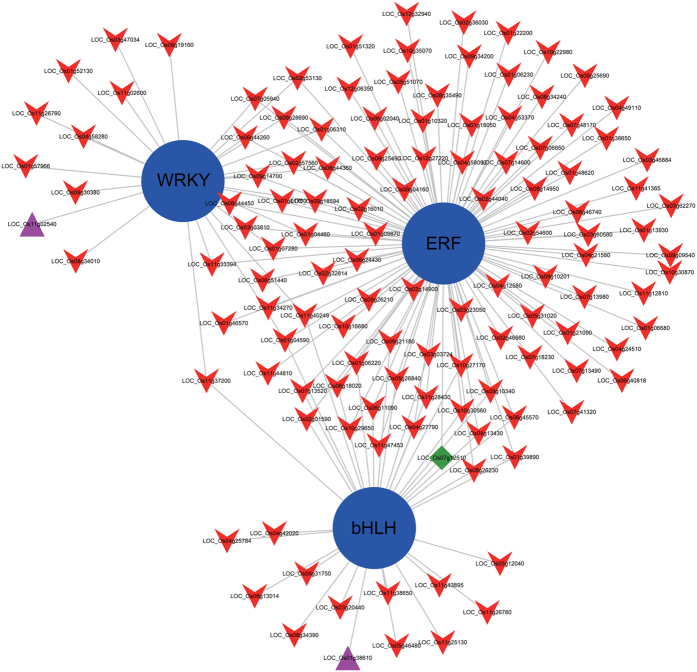
Gene network represents the transcription factors (TFs) involved in their putative transcription factor binding sites (TFBSs) in promoters of 166 DEGs in HuHan2B. Three TF families were colored blue and considered transcriptional activators in ABA-inducible gene expression under drought stress in plants. Among the TF families, two TFs colored pink and one TF colored green were found to have TFBS involved in the transcriptional regulation of their promoters.

To understand the cooperative relationship between transcription factors and DEGs, we identified transcription factor family and transcription factor binding sites (TFBS) within 3 Kb upstream of the promoter sequence to transcription start site (TSS) among 166 DEGs. We observed that 117 DEGs were predicted with TFBS in HuHan2B and may potentially represent the binding sites of 3 TF families, as well as ERF, WRKY, and bHLH (Fig. 6). Strikingly, the overrepresented interaction between TFs and TFBS of regulated genes can be essential in enhancing drought resistance in HuHan2B. Next, based on the results of specific inherited allele-related genes in HuHan2B, we found 36 genes with SNPs in the genebody or in proximity of the promoter, and 28 genes were involved in the regulatory network between TFs and target genes, which revealed that the allelic-influencing genes have a high efficiency (28/36 specific inherited allele-related DEGs in HuHan2B) in response to drought. The majority of 28 genes with specific inherited alleles from parents other than HanFengB exhibited the same parent origin from the deduced genomic constitution (Table S6). The higher enrichment of inherited allele-related genes in network revealed that mutual cross-regulation among groups of TFs enhanced the drought resistance inHuHan2B.

**Figure 7:**
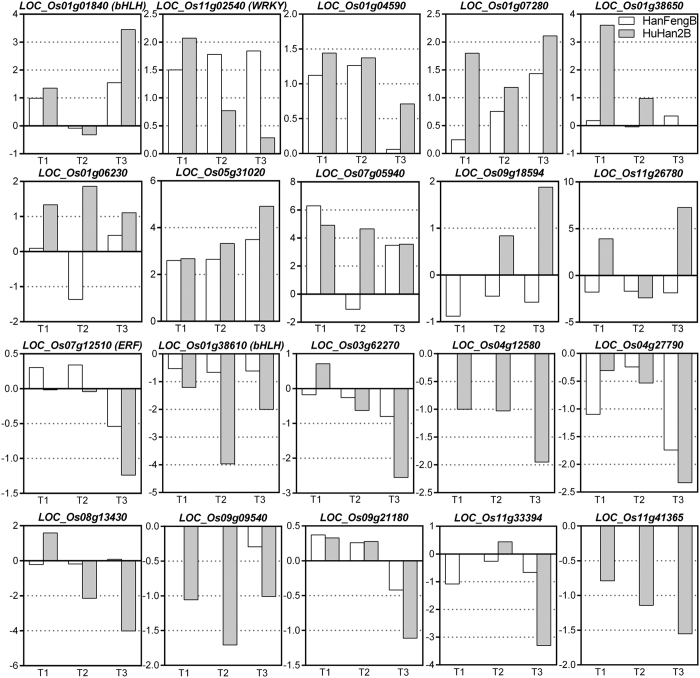
Comparison of changes of TF and TFBS genes with inherited alleles in the FPKM(drought stress)/FPKM(well watered) ratio in HuHan2B and HanFengB at the T1-T3 time points. The values represent the log2 fold change of FPKM expression values.

Furthermore, our data showed that drought stress caused considerable alterations in expression of TFs and TFBS genes, even at all three time points. By comparing the fold changes of expression between drought stress and control conditions, consistent expression trends were observed for TFs and part of allelic-influencing TFBS genes (Fig. 7). For one of bHLH and WRKY TFs, their expressions showed a closer association with up-regulated TFBS genes, whereas one of bHLH and ERF TFs were down-regulated as similar as possible to the expression pattern of allelic-influencing TFBS genes (Fig. 7). A large number of information demonstrated the involvement of ERF, WRKY and bHLH in receptor-mediated processes in response to drought stress. For many TFBS genes, drought response activity was reported for the induction of the adaptive processes in a drought environment. Among these genes in the network, we found that a protein synthesis related gene eRF1-1 (LOC_Os05g31020), a member of the eukaryotic release factor 1 (eRF1) family, was highly induced by drought stress. Induction of eRF1-1 upon drought stress was much higher in HuHan2B than that in HanFengB, especially at the T2 and T3 time points (Fig. 7). Interestingly, the gradually accumulating expression of eRF1 indicated its potential role in mediating rice responses to drought stress through its function in translation termination. Such versatility in TF functions contributed to the modification of the temporal and spatial expression patterns of specific stress-responsive genes; these TFs were an important part of the plant drought stress response. Notably, differential expression patterns showed more evidence for prevalent specific inherited alleles that contribute to greater expression differences between HuHan2B and HanFengB (Fig. 7). Additionally, the induction of stress-related genes primarily occurred at the transcriptional level. TFs represented the key molecular switches and activated/suppressed the downstream target genes by the regulatory elements in the network of TFs and TFBS genes, and a greater change in the differential expression suggested that the regulation of transcription was more influenced by inherited variations in TFBS genes in HuHan2B. Therefore, the genetic constitution inherited from parents contributed to transform major-effect genes into offspring HuHan2B, which were congregated in manner of interacting network by breeding.

## Discussion

Plant backcross breeding has become a widely used approach in diverse crop species to incorporate one or a few traits into an adapted or elite variety^20^,^21^. HuHan2B maintained a yield potential to the recurrent parental HanFengB, but showed better drought resistance in fields than HanFengB^17^. However, interpreting such phenotypic differences is often difficult due to a high similarity in morphological characteristics and genetic constitution. Instead of seeking the individual loci, genomic constitution and transcriptomic analyses were used. Our results provided further evidences for the assumption that multiple drought-responsive genes were inherited and transformed into offspring HuHan2B in manner of gene network.

Based on the genotyping by resequencing, the genetic similarity between HuHan2B and HanFengB is 84%, which was similar to that in previous studies using SSR markers^17^. Reconstruction of the pedigree breeding history suggested that HuHan2B inherited 62.39% of its genomic content from the recurrent parent HanFengB. The genomic recombination retained larger chromosomal regions of its grandparents, MaWanNuo and IRAT109 (Fig. 1).

Drought resistance is a quantitative trait, implying many genes are involved in activating a series of complicated regulatory mechanisms to enhance drought resistance^6^. Transcriptomic performance under drought stress and control conditions across different drought stages led us to several important conclusions. First, genetic variation of rice varieties contributed to more effects on differences of gene expression levels than that of drought stress treatment. PC1 accounted for ~51% of the observed variation and corresponded to species types, which was reflected by the main genomic differences between HuHan2B and the recurrent parent HanFengB (Fig. 4). This was not surprising because the preferential selection for drought resistance was carried out during HuHan2B breeding; in addition, the agricultural traits that were most significantly improved were originally drought resistance traits selected from the grandparents^17^. This result also suggested that genomic content inherited to adapt to drought stress accumulated in the genome of HuHan2B during pedigree breeding.

Second, our results indicated that the regulative mechanism of response to drought stress should be largely effective in HuHan2B. The number of common DEGs of up/down-regulated genes was 448 between HuHan2B and HanFengB, which accounts for approximately one third of the DEGs in each variety under drought stress. Thus, different ways were adapted by HuHan2B and HanFengB when responding to drought stress. Interestingly, we observed that the majority of DEGs in HuHan2B were regulated by transcription factors. Furthermore, the specific inherited allele-related genes in HuHan2B were primarily involved in the regulatory network of TFs and target genes (Fig. 6). This suggested that the signal transduction pathway remains the primary mechanism regulating drought resistance. In contrast, the enriched oxidation-reduction process in HanFengB indicated that oxidative damage to cellular components were rapidly worsening, which frequently triggers programmed cell death (PCD)^22^. Highly cooperative regulation may be more important for the response of the rice plant to a drought environment than an enhanced oxidation-reduction process.

Third, it is striking that the manner of response of the DEG modules can be tuned to enhance cooperative regulation by cross-talk between TFs and TFBS genes due to specific inherited alleles in HuHan2B. This property will provide a new perspective in reshaping the elite variety and contrasts with transgenic rice using more donor genes to control drought resistance. We noted that considerable alterations in the regulatory network of TFs and target genes were observed at all three time points and that the fold changes of expression in HuHan2B were far more extensive than those in HanFengB (Fig. 7). These results may indicate that the cooperative regulation of TFs plays crucial roles in response to drought signals^23^. Our analysis did not include the effects of inherited allelic influencing genes under well watered conditions, which would be of interest to further studies. Differential regulation may slightly delay responses to drought stress; thus, DEGs under well watered conditions could affect response dynamics to the duration and the intensity of the stress treatment.

Finally, the findings of the genomic constitution and transcriptomic analyses showed the amounts of useful genetic variation for WDR breeding. While selecting excellent valuable agronomic traits for WDR, we congregated more genomic constitution related to drought-responsive genes into offspring. The results revealed that the inherited allele-related genes were transformed into HuHan2B in manner of network to enhance drought resistance by pedigree breeding. Thus, exploiting the potential biological function in inherited allelic-influencing DEGs using pedigree breeding will be of great importance in improving the selection efficiency and overall genetic gain of multiple complex traits. However, considerable genetic gains in complex traits remain a challenge due to the involvement of multiple pathways. Genetic determination thus presents further challenges when considering yield and other economic traits in crop breeding.

## Materials and Methods

### Plant materials

Seeds of five rice varieties, MaWanNuo, IRAT109, HuHan3, HanFengB and HuHan2B, were collected from Shanghai Agrobiological Gene Center, China. HuHan3 was a drought-resistant type that was bred from three parental lines, Chinese local upland rice variety MaWanNuo from Hubei Province, African upland variety IRAT109, and American high-quality rice variety P77. HanFengB was a maintainer of the japonica hybrid rice combination Hanyouxiangqing that has been commercialized in Shanghai for decades. HuHan2B was a drought-resistant maintainer developed from backcross breeding using HuHan3 as the donor of drought resistance and HanFengB, susceptible to drought, as the recurrent parent (Fig. 1a)^17^.

### Genome resequencing and genotyping of HuHan2B pedigree varieties

Genomic DNA from healthy leaves was extracted using the CATB procedure^24^. Following quality assessment, random fragmenting and size selection between 350 and 500 bp, the genomic DNA was subjected to Illumina paired-end sequencing with lengths of 100 or 126 bp using the Illumina HiSeq 2000 or HiSeq 2500 system, respectively, by the Suzhou BeisiPai Biological Science & Technology Co.Ltd.

All reads were filtered with skewer v0.1.123 to obtain correct paired reads using a minimum read length of 70 and the lowest mean quality of 20. We then removed low quality reads with the NGS QC Toolkit v2.3.3 and obtained high-quality and adaptor-free reads (clean reads) using a cutoff read length of 70% and quality score of 20. Approximately 0.5 billion clean reads from the five rice varieties with average coverage depths from 5X to 50X were mapped to the annotated reference genome of Nipponbare (MSU Rice Genome Annotation Project Release 7.0)^25^ by the gap-enabled Burrows Wheeler aligner (BWA)^26^, allowing up to 4% mismatches and 1 gap. As varieties were analyzed together, SNPs were called with SAMtools^27^ and GATK^28^,^29^, requiring a mapping quality of 70 and at least 3 reads per allele.

### SNP validation with Illumina RiceSNP60 array

SNPs were assayed using the RiceSNP60 array, which contains approximately 51,000 evenly distributed SNP markers^13^. Four rice varieties, including IRAT109, HuHan3, HanFengB and HuHan2B, were prepared and assayed by the Life Science and Technology Center, China National Seed Group Co., LTD (Wuhan, China), according to the Infinium HD Assay Ultra Protocol (http://www.illumina.com/). The discordant rate percentages were calculated by dividing the number of different SNPs by a common set of homozygosis SNP loci between the RiceSNP60 SNP array and the SNP calling from resequencing.

### Genetic similarities and reconstructions of the HuHan2B breeding history

Genetic similarities between the genotyped HuHan2B and its pedigree breeding varieties were calculated using the genetic similarity index (π) proposed by Nei and Li^30^. To enhance our understanding of genetic diversity and relatedness among varieties of HuHan2B pedigree breeding, we reconstructed the breeding history of HuHan2B using the method of Lai *et al.*^31^. In brief, scanning the diversity-decreased level was used to estimate the source of genomic constitutes for all five rice varieties in one region of the reference genome. The workflow was as follows^31^: (1) Dpair was used to estimate the diversity level of two samples for each parent and descendant pairs. (2) Darv was used to estimate the average diversity level for all five samples. (3) (Darv − Dpair)/Darv was defined as the diversity-decreased level with sliding windows based on the SNP conservation. Then, using a proper approach for bimodal distributions, we defined high confidence haplotype blocks of genomic regions that are descended from different parents, which were assessed as descending from a particular parental germplasm. Moreover, uncertain regions (on the boundary between two types) were equally apportioned into the two adjacent blocks. Finally, genomic regions descended from different parents were set to different colors according to the type of haplotype block.

### Field experiment of drought stress treatments

Drought resistance of HuHan2B and HanFengB, together with 30 other varieties, was evaluated in an experiment with split randomized block design with three replications. Each blot had 100 plants arranged in 10 rows with 18 cm space between rows and 16 cm space between plants. Presoaked seeds were sown in nursery on May 5, 2014. Seedlings were transplanted on June 15. Irrigation was withdrawn on July 16 initiate the drought stress in one split area (blocks with drought stress treatment). Leaf sampling for RNA-Seq was made on the 20th day after water withholding (T1) when soil moisture declined from 23.1% to 10.0%. Two more sampling time points (T2 and T3) were on the 25^th^ and 37^th^ day, respectively. Irrigation was restored on the 38^th^ day. Leaf desiccation were recorded on August 26 and represented as the proportion of the number of desiccated leaves to total leaf number.

### RNA-Seq and differential expression analysis

The leaf tissues of both HuHan2B and HanFengB grown in the well-irrigated and drought stress treatments were harvested at 13:00–14:00, frozen in liquid N2 immediately and stored at −80 °C until RNA extraction. Each sample was taken from three plants and pooled for biological replicates. Total RNA was respectively isolated from the leaf tissues of three plants, and equivalent amounts of RNA were combined into one sample pool for sequencing. A total of 12 RNA samples, from two varieties, two treatments and three time points were sequenced by Shanghai Major Bio-Pharm Technology Co.Ltd, and the 126 bp paired-end reads with non-strand-specific RNA sequences were generated using the Illumina HiSeq2500 system in one flow cell with eight lanes producing 20 million reads per sample.

After stringent data cleaning and quality checks, these sequences were mapped against the Nipponbare reference genome (MSU 7.0) using TopHat v2.0.10^32^ software with Bowtie2 v2.2.5^33^. Then, we used Cufflink to normalize each gene transcript expression by fragment per kilobase of exon model per million mapped reads (FPKM) and Cuffdiff to determine significant changes in gene transcript expression between each condition. Genes with the criteria of fold change >2 and a p-value < 0.05 were considered statistically significant.

To exclude the impact of developmental genes and to obtain a relatively consistent control background, we used Cuffdiff to determine and remove the differential expression between each time point under the well watered condition. We could then determine the DEGs between each time point after the drought stress treatment that played a large role in response to drought stress in HuHan2B and HanFeng, respectively. The total DEGs were collected between well-irrigated and drought stress conditions at T1, between drought stress conditions at T1 and T2, and between drought stress conditions at T2 and T3. Principal component analysis on the entire transcriptome dataset was performed using R software (3.2.3).

### Prediction of transcription factor binding sites

We found TFs from the annotated resource PlantTFDB (http://planttfdb.cbi.pku.edu.cn/)^34^. While TFs act at the DNA level by binding to cis-regulatory elements of genes, we used the JASPAR database to identify binding-site motifs in promoters of DEGs, a sequence 3,000 bp upstream of the transcription start site (TSS) in the MSU annotation of the genome data^35^. The interaction networks for each TF family and TFBS gene were visualized using an interaction graph by Cytoscape^36^.

### Data availability

The raw data of whole-genome resequencing has been deposited to the NCBI Sequence Read Archive (SRA) with the Bioproject number PRJNA260762. The RNA-Seq experiment numbers for the 12 pools from HuHan2B and HanFengB were in Bioproject PRJNA306542 with experiments from SRX1521275 to SRX1521286.

## Additional Information

**How to cite this article**: Wei, H. *et al.* Genetic determination of the enhanced drought resistance of rice maintainer HuHan2B by pedigree breeding. *Sci. Rep.* **6**, 37302; doi: 10.1038/srep37302 (2016).

**Publisher’s note**: Springer Nature remains neutral with regard to jurisdictional claims in published maps and institutional affiliations.

## Supplementary Material

Supplementary Table S1

Supplementary Table S2

Supplementary Table S3

Supplementary Table S4

Supplementary Table S5

Supplementary Table S6

## Figures and Tables

**Table 1:** Genomic resequencing and analysis statistics of the HuHan2B pedigree breeding varieties relative to the Nipponbare reference genome.

Samples	Raw reads	Clean reads	Fragment size (bp)	Sequencing depth (fold)	Mapping rate	Genome coverage ≥(15x)	Coding region coverage		
							≥1x	≥5x	≥15x
MaWanNuo	104,287,224	97,174,088	336.56	31.99	97.87%	92.51%	99.26%	99.00%	95.20%
IRAT109	110,478,864	110,190,140	434.99	28.07	95.51%	82.32%	97.53%	96.45%	91.40%
HuHan3	24,365,640	20,627,646	216.79	5.3	96.50%	1.02%	76.30%	40.50%	0.31%
HanFengB	97,304,416	90,632,804	332.87	30	98.38%	92.81%	99.47%	99.24%	95.72%
HuHan2B	164,533,962	157,819,532	327.02	51.7	97.35%	94.49%	99.16%	99.04%	98.71%

**Table 2:** Genetic similarity indexes (1 − π) between five varieties of HuHan2B pedigree breeding.

Samples	MaWanNuo	IRAT109	HuHan3	HanFengB	HuHan2B
MaWanNuo	1				
IRAT109	0.16	1			
HuHan3	0.48	0.53	1		
HanFengB	0.86	0.21	0.47	1	
HuHan2B	0.82	0.14	0.52	0.84	1
